# Supplementary material for: Double burden of gestational diabetes and pregnancy-induced hypertension in Ethiopia: A systematic review and meta-analysis of observational studies
Source: PLoS One. 2024 Oct 2;19(10):e0311110. doi: 10.1371/journal.pone.0311110 (PMC11446441; doi:10.1371/journal.pone.0311110)
Supplement: S2 Table — (PDF) [file pone.0311110.s002.pdf]

Critical Appraisal

a) Critical appraisal was conducted by three independent reviewers (EGA, EKG, and DY) between 18/05/2023 and 24/05/2023 using a standardized critical appraisal instrument developed by JBI for systematic reviews of observational studies

Critical Appraisal of Eligible Analytical Cross-Sectional Study (n=8)

Study	Q1	Q2	Q3	Q4	Q5	Q6	Q7	Q8	Ass.
Asefa F, Hunde A, Siraneh Y. 2020.	N	NA	NA	NA	NA	NA	NA	NA	0

Overall appraisal: ***Include*** _________ ***Exclude*** ***__X____*** ***Seek further info*** ☐

Comments (Including reason for exclusion): ***We excluded the study by Asefa F, Hunde A, and Siraneh Y, 2020, titled 'Outcome of Hypertensive Disorders of Pregnancy and Associated Factors Among Pregnant Women Admitted to Jimma University Medical Center, Southwest Ethiopia,' because the outcome variables did not meet our inclusion criteria. The study focused on hypertensive disorders of pregnancy and associated factors without addressing the burden among mothers with GDM. The published study can be accessed at the following*** ***URL: http://198.1.99.189/index.php/ejrh/article/view/397.***

Study	Q1	Q2	Q3	Q4	Q5	Q6	Q7	Q8	Ass.
Boka A, Nigatu D. 2019.	Y	Y	Y	Y	Y	Y	Y	U	7

Overall appraisal: ***Include*** ***____X_____Exclude*** ☐ ***Seek further info*** ☐

Comments (Including reason for exclusion):

Study	Q1	Q2	Q3	Q4	Q5	Q6	Q7	Q8	Ass.
Eshetu B, Sintayehu Y, Mekonnen B, Daba W. 2019.	Y	Y	Y	Y	N	Y	Y	N	6

Overall appraisal: *Include* ___*X*___*Exclude* ☐ *Seek further info* ☐

Comments (Including reason for exclusion):

Study	Q1	Q2	Q3	Q4	Q5	Q6	Q7	Q8	Ass.
Andarge RB, Anshebo AA, Halil HM, Kebede BA, Abdo RA. 2020.	Y	Y	Y	Y	N	U	Y	Y	6

Overall appraisal: *Include* ___*X*___*Exclude* ☐ *Seek further info* ☐

Comments (Including reason for exclusion):

Study	Q1	Q2	Q3	Q4	Q5	Q6	Q7	Q8	Ass.
Ayalew AF, Bantie GM, Kassa TB, Woya AA. 2019.	Y	Y	U	Y	N	U	Y	Y	5

Overall appraisal: *Include* ___*X*___*Exclude* ☐ *Seek further info* ☐

Comments (Including reason for exclusion):

Study	Q1	Q2	Q3	Q4	Q5	Q6	Q7	Q8	Ass.
Baynesagn SA. 2018.	N	NA	NA	NA	NA	NA	NA	NA	0

Overall appraisal: *Include* ________*Exclude* ___X____ *Seek further info* ☐

Comments (Including reason for exclusion): ***We excluded the study by Baynesagn SA, 2018, titled 'Prevalence and Associated Factors of Pregnancy-Induced Hypertension Disorder Among Women Delivered in Gelemso General Hospital, Oromia Regional State, Eastern Ethiopia,' because the outcome variables did not meet our inclusion criteria. The paper only reported the prevalence of pregnancy-induced hypertension without addressing its burden among mothers with GDM. As the paper was unpublished, here is the URL:*** ***http://ir.haramaya.edu.et/hru/bitstream/handle/123456789/3219/Semagn%20Alemnew.pdf?sequence=1***.

Study	Q1	Q2	Q3	Q4	Q5	Q6	Q7	Q8	Ass.
Belay AS, Wudad T. 2019.	N	NA	NA	NA	NA	NA	NA	NA	0

Overall appraisal: *Include* ________*Exclude* ___X____ *Seek further info* ☐

Comments (Including reason for exclusion): ***We excluded the study by Belay AS and Wudad T, 2019, titled 'Prevalence and Associated Factors of Pre-Eclampsia Among Pregnant Women Attending Antenatal Care at Mettu Karl Referral Hospital, Ethiopia: A Cross-Sectional Study,' because the outcome variables did not meet our inclusion criteria. The study focused on the prevalence and associated factors of pre-eclampsia but did not address its burden among mothers with GDM. The published study can be accessed at the following*** *URL:* ***https://link.springer.com/article/10.1186/s40885-019-0120-1***.

Study	Q1	Q2	Q3	Q4	Q5	Q6	Q7	Q8	Ass.
Firisa W, Onsongo L, Mugo J. 2021.	Y	Y	Y	N	U	Y	Y	Y	6

Overall appraisal: *Include* ___X____*Exclude* ☐ *Seek further info* ☐

Comments (Including reason for exclusion):

Critical Appraisal of Eligible Case Control Study (n=9)

Study	Q1	Q2	Q3	Q4	Q5	Q6	Q7	Q8	Q9	Q10	Ass.
Debele BS, Endris BS, Mengistu YG, Haider JA. 2023.	Y	Y	N	N	N	N	Y	Y	Y	Y	6

Overall appraisal: ***Include ____X____Exclude*** □ ***Seek further info*** □

Comments (Including reason for exclusion):

Study	Q1	Q2	Q3	Q4	Q5	Q6	Q7	Q8	Q9	Q10	Ass.
Duko B. 2021.	Y	Y	Y	Y	U	N	N	Y	Y	N	6

Overall appraisal: ***Include ____X____Exclude*** □ ***Seek further info*** □

Comments (Including reason for exclusion):

Study	Q1	Q2	Q3	Q4	Q5	Q6	Q7	Q8	Q9	Q10	Ass.
Haymanot T, Abebe E, Mistire W. 2020.	Y	Y	U	Y	Y	N	N	Y	Y	Y	7

Overall appraisal: ***Include ____X____Exclude*** □ ***Seek further info*** □

Comments (Including reason for exclusion):

Study	Q1	Q2	Q3	Q4	Q5	Q6	Q7	Q8	Q9	Q10	Ass.
Hinkosa L, Tamene A, Gebeyehu N. 2020.	N	NA	NA	N	NA	NA	NA	NA	NA	NA	0

Overall appraisal: *Include* ________ *Exclude* ___X____ *Seek further info* □

Comments (Including reason for exclusion): ***We excluded the study by Hinkosa L, Tamene A, and Gebeyehu N, 2020, titled 'Incidence and Predictors of Pre-Eclampsia Among Pregnant Women Attending Antenatal Care at Debre Markos Referral Hospital, North West Ethiopia: Prospective Cohort Study,' as the outcome variables did not meet our inclusion criteria. The paper focused on the incidence and predictors of pre-eclampsia without addressing its impact among mothers with GDM. The published study can be accessed at the following*** *URL:* https://chatgpt.com/c/66e82045-9700-800a-9549-9d73d30597ff.

Study	Q1	Q2	Q3	Q4	Q5	Q6	Q7	Q8	Q9	Q10	As.
Kahsay HB, Gashe FE, Ayele WM. 2018.	Y	Y	Y	Y	Y	N	N	U	Y	Y	7

Overall appraisal: *Include* ___X____ *Exclude* □ *Seek further info* □

Comments (Including reason for exclusion):

Study	Q1	Q2	Q3	Q4	Q5	Q6	Q7	Q8	Q9	Q10	Ass.
Katore FH, Gurara AM, Beyen TK. 2021.	Y	Y	Y	N	Y	N	N	Y	Y	N	6

Overall appraisal: *Include* ___X____ *Exclude* □ *Seek further info* □

Comments (Including reason for exclusion):

Study	Q1	Q2	Q3	Q4	Q5	Q6	Q7	Q8	Q9	Q10	Ass.
Kidane R, Eshete T, Sintayehu T, Belachew T. 2022.	Y	Y	Y	Y	Y	U	N	Y	Y	Y	8

Overall appraisal: Include ___X___Exclude ☐ Seek further info ☐

Comments (Including reason for exclusion):

Study	Q1	Q2	Q3	Q4	Q5	Q6	Q7	Q8	Q9	Q10	Ass.
Wakwoya EB, Fita FU. 2018.	Y	Y	Y	N	U	N	N	Y	Y	Y	6

Overall appraisal: Include ___X___Exclude ☐ Seek further info ☐

Comments (Including reason for exclusion):

Study	Q1	Q2	Q3	Q4	Q5	Q6	Q7	Q8	Q9	Q10	Ass.
Jikamo B, Adefris M, Azale T, Alemu K.	N	NA	NA	NA	NA	NA	NA	NA	NA	NA	0

Overall appraisal: Include ________Exclude ___X___ Seek further info ☐

Comments (Including reason for exclusion): ***We excluded the study by Jikamo B, Adefris M, Azale T, and Alemu K, titled 'Risk Factors for Preeclampsia and Eclampsia in Sidama Region, Southern Ethiopia: A Nested Case-Control Study,' because the outcome variables did not meet our inclusion criteria. The study focused on risk factors for preeclampsia and eclampsia but did not address the burden among mothers with GDM. The published study can be accessed at the following*** *URL:* ***https://www.researchgate.net/profile/Birhanu-Bago/publication/363504069_Risk_factors_for_preeclampsia_and_eclampsia_in_Sidama_region_southern_Ethiopia_a_nested_case-control_study/links/634eb38a12cbac6a3ed731b2/Risk-factors-for-preeclampsia-and-eclampsia-in-Sidama-region-southern-Ethiopia-a-nested-case-control-study.pdf.***

Critical Appraisal of Eligible Cohort Study (n=4)

Study	Q1	Q2	Q3	Q4	Q5	Q6	Q7	Q8	Q9	Q10	Q11	Ass.
Birhanu MY, Temesgen H, Demeke G, Assemie MA, Alamneh	N	NA	NA	NA	NA	NA	NA	NA	NA	NA	NA	0

AA, Desta M, et al. 2020.												

Overall appraisal: ***Include ________Exclude ___X_____ Seek further info ☐***

Comments (Including reason for exclusion): We excluded the study by Birhanu MY, Temesgen H, Demeke G, Assemie MA, Alamneh AA, Desta M, et al., 2020, titled 'Incidence and Predictors of Pre-Eclampsia Among Pregnant Women Attending Antenatal Care at Debre Markos Referral Hospital, North West Ethiopia: A Prospective Cohort Study,' as the outcome variables did not meet our inclusion criteria. The study focused on the incidence and predictors of pre-eclampsia, without addressing its burden among mothers with GDM. The published study can be accessed at the following URL:

https://www.tandfonline.com/doi/full/10.2147/

**Study**	**Q1**	**Q2**	**Q3**	**Q4**	**Q5**	**Q6**	**Q7**	**Q8**	**Q9**	**Q10**	**Q11**	**Ass.**
Muche AA, Olayemi OO, Gete YK. 2020.	Y	Y	Y	U	N	Y	Y	Y	U	Y	Y	8

Overall appraisal: ***Include ___X____Exclude ☐ Seek further info ☐***

Comments (Including reason for exclusion):

**Study**	**Q1**	**Q2**	**Q3**	**Q4**	**Q5**	**Q6**	**Q7**	**Q8**	**Q9**	**Q10**	**Q11**	**Ass.**
Welesemayat ET, Taye G, Seid Y, Gufue ZH. 2020.	Y	Y	Y	N	N	U	Y	Y	Y	Y	Y	8

Overall appraisal: ***Include ___X____Exclude ☐ Seek further info ☐***

Comments (Including reason for exclusion):

**Study**	**Q1**	**Q2**	**Q3**	**Q4**	**Q5**	**Q6**	**Q7**	**Q8**	**Q9**	**Q10**	**Q11**	**Ass.**
Wolka E, Deressa W, Reja A. 2022.	Y	Y	Y	N	N	Y	Y	Y	U	Y	Y	8

Overall appraisal: ***Include*** ***____X_____Exclude*** *☐* ***Seek further info*** *☐*

Comments (Including reason for exclusion):
