# Supplementary material for: Double burden of gestational diabetes and pregnancy-induced hypertension in Ethiopia: A systematic review and meta-analysis of observational studies
Source: PLoS One. 2024 Oct 2;19(10):e0311110. doi: 10.1371/journal.pone.0311110 (PMC11446441; doi:10.1371/journal.pone.0311110)
Supplement: S3 Table — (PDF) [file pone.0311110.s003.pdf]

Extracted Studies

a) Data extraction was conducted by three independent reviewers (EGA, EKG, and DY) between May 25 and June 03, 2023 using a standardized data extraction tool developed by JBI, and the data were subsequently sorted.

b) We confirm that all studies included in the review were eligible based on predefined inclusion criteria.

c) All data extracted for this systematic review and meta-analysis, including sample size, region, study design, mean age, standard deviation, and prevalence, with the studies quality assessment are essential for replicating the analyses.

Characteristics of Included Studies

Study	Publication year	Region	Study design	Quality Ass.	Sample	Mean age (SD)	Prevalence (%)
Andarge	2020	SNNPR	Cross-Sectional	6	242	27.70 (4.00)	2.07
Ayalew	2019	Amhara	Cross-Sectional	5	193	27.73 (4.30)	3.63
Duko	2021	SNNPR	Case Control	6	283	26.10 (5.40)	3.53
Firisa	2021	Addis Ababa	Cross-Sectional	6	297	28.98 (9.85)	5.72
Haymanot	2020	Amhara	Case Control	7	200	29.07 (6.39)	4.00
Kahsay	2018	Tigray	Case Control	7	330	26.94 (5.73)	2.12
Katore	2021	Oromia	Case Control	6	302	26.29 (4.70)	0.99
Kidane	2022	Oromia	Case Control	8	312	29.08 (6.42)	6.41
Welesemayat	2020	Tigray	Cohort	8	476	33.53 (5.83)	2.10
Boka	2019	Oromia	Cross-Sectional	7	346	26.10 (5.40)	13.58
Debele	2023	Addis Ababa	Case Control	6	128	29.41 (4.84)	9.38
Eshetu	2019	Addis Ababa	Cross-Sectional	6	346	30.80 (4.70)	13.58
Muche	2020	Amhara	Cohort	8	694	27.67 (5.03)	2.16
Wakwoya	2018	Oromia	Case Control	6	1834	25.60 (4.80)	8.51
Wolka	2022	SNNPR	Cohort	8	408	27.54 (4.85)	3.19
