# Supplementary material for: Double burden of gestational diabetes and pregnancy-induced hypertension in Ethiopia: A systematic review and meta-analysis of observational studies
Source: PLoS One. 2024 Oct 2;19(10):e0311110. doi: 10.1371/journal.pone.0311110 (PMC11446441; doi:10.1371/journal.pone.0311110)
Supplement: S4 Table — (PDF) [file pone.0311110.s004.pdf]

Std_Eff	Coefficient	Std. err.	T	P>t	[95% confidence interval]	
Slope	.2251507	.4374444	0.51	0.615	-.7198903	1.170192
Bias	.9717371	.4687842	2.07	0.059	-.0410096	1.984484
