# Supplementary figures and images for: Double burden of gestational diabetes and pregnancy-induced hypertension in Ethiopia: A systematic review and meta-analysis of observational studies
Source: PLoS One. 2024 Oct 2;19(10):e0311110. doi: 10.1371/journal.pone.0311110 (PMC11446441; doi:10.1371/journal.pone.0311110)

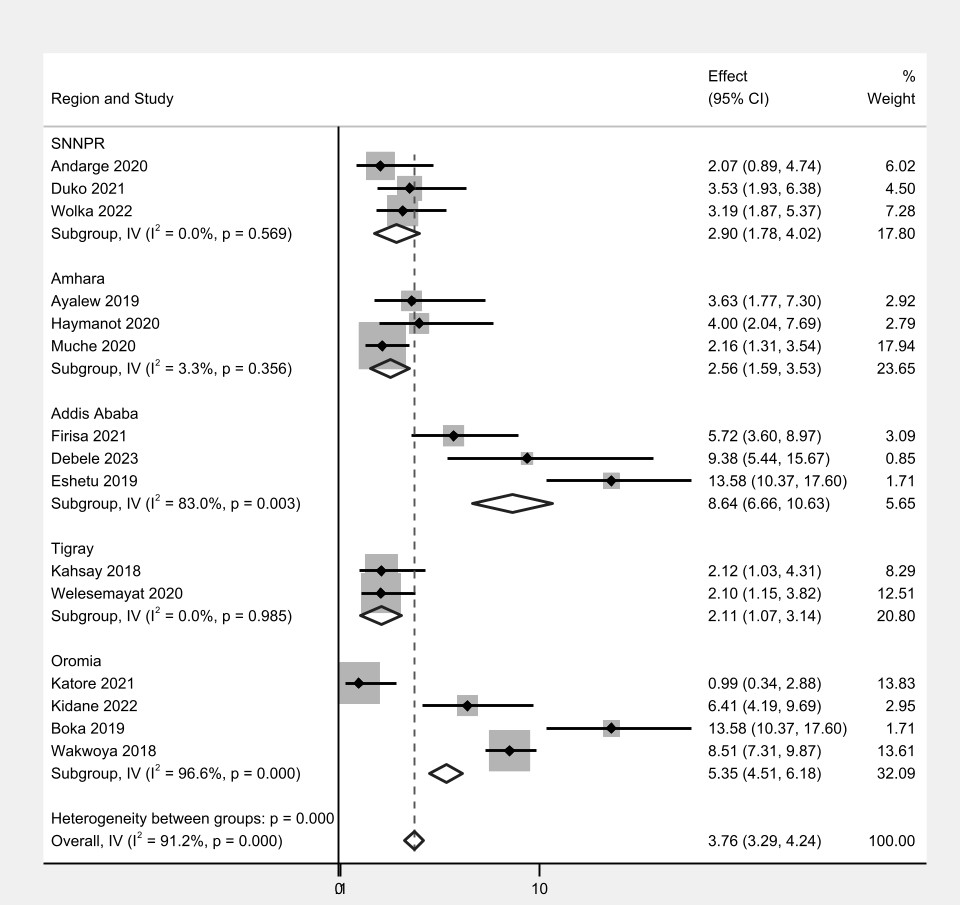

Supplement: S1 Fig — (TIF) [file pone.0311110.s005.tif]

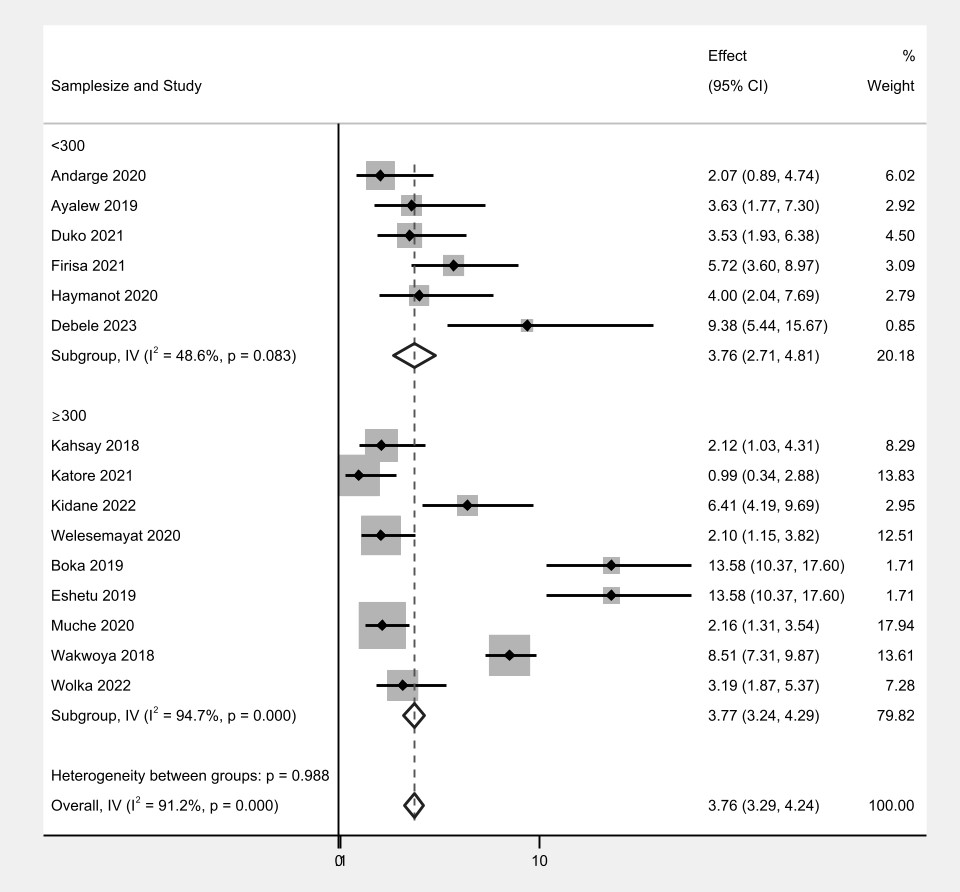

Supplement: S2 Fig — (TIF) [file pone.0311110.s006.tif]

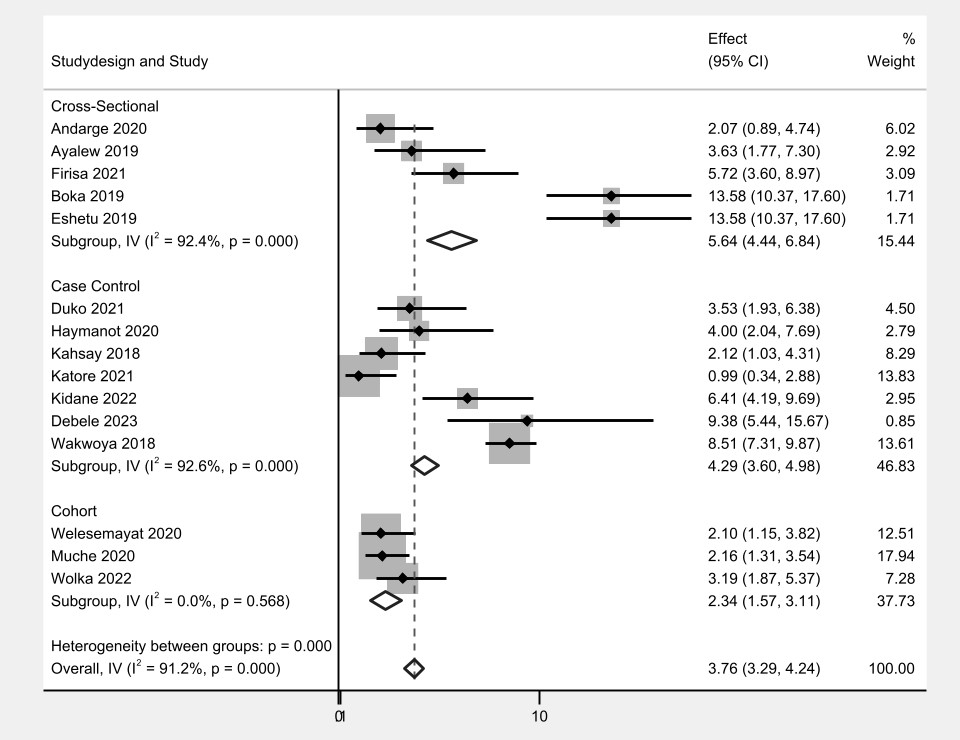

Supplement: S3 Fig — (TIF) [file pone.0311110.s007.tif]

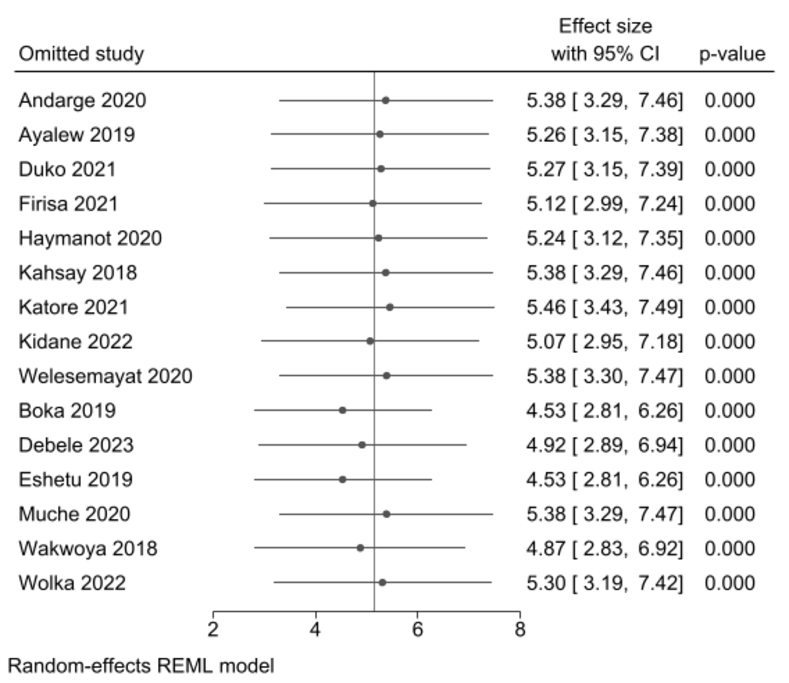

Supplement: S4 Fig — (TIF) [file pone.0311110.s008.tif]

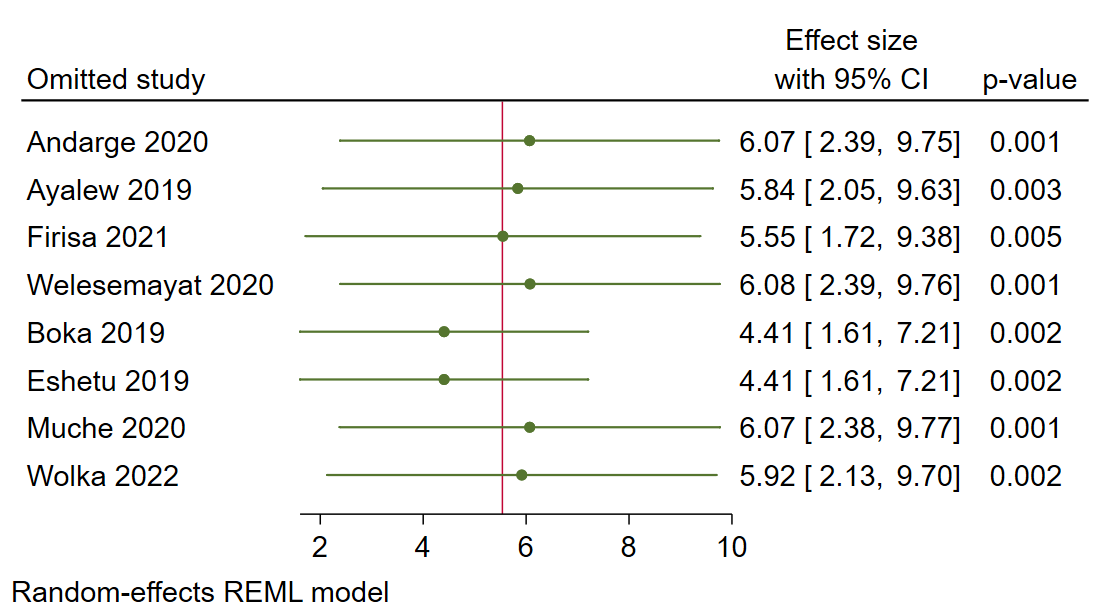

Supplement: S5 Fig — (TIF) [file pone.0311110.s009.tif]

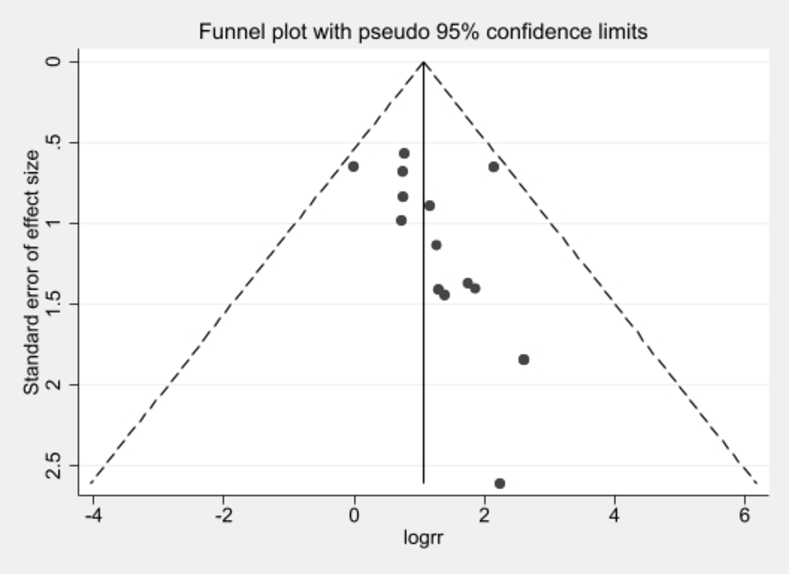

Supplement: S6 Fig — (TIF) [file pone.0311110.s010.tif]
