# Supplementary material for: Double burden of gestational diabetes and pregnancy-induced hypertension in Ethiopia: A systematic review and meta-analysis of observational studies
Source: PLoS One. 2024 Oct 2;19(10):e0311110. doi: 10.1371/journal.pone.0311110 (PMC11446441; doi:10.1371/journal.pone.0311110)
Supplement: S1 Appendix — (PDF) [file pone.0311110.s011.pdf]

(Performed during March 15 – May 14, 2023)

Search strategy of Gestational Diabetes Mellitus and Pregnancy Induced Hypertension: Co-Occurrence and Correlation among Pregnant Women in Ethiopia: A Systematic Review and Meta- Analysis

PubMed

No.		Condition (Co); Pregnancy Induced Hypertension Diabetes, Gestational	Context (Co); Ethiopia	Population (Po); Pregnant Women
1.	**Mesh term**	**Hypertension, Pregnancy-Induced**	**Ethiopia**	**pregnant women**
2.		**Diabetes, Gestational**		
3.	**Entry terms**	Hypertension, Pregnancy Induced	Federal Democratic Republic of Ethiopia	Pregnant Woman
4.		Pregnancy-Induced Hypertension		Women, Pregnant
5.		Pregnancy Induced Hypertension		Woman, Pregnant
6.		Hypertensions, Pregnancy Induced		
7.		Induced Hypertension, Pregnancy		
8.		Induced Hypertensions, Pregnancy		
9.		Gestational Hypertension		
10.		Hypertension, Gestational		
11.		Transient Hypertension, Pregnancy		
12.		Hypertension, Pregnancy Transient		
13.		Pregnancy Transient Hypertension		
14.		Eclampsia		
15.		Pre-Eclampsia		
16.		Preeclampsia		
17.		Hypertensive Disorders, Pregnancy		
18.		Diabetes, Pregnancy-Induced		
19.		Diabetes, Pregnancy Induced		
20.		Pregnancy-Induced Diabetes		
21.		Gestational Diabetes		

22.		Diabetes Mellitus, Gestational		
23.		Gestational Diabetes Mellitus		
24.		**((((((((((((((((Hypertension, Pregnancy-Induced[MeSH Terms]) OR (Hypertension, Pregnancy Induced[Title/Abstract])) OR (Pregnancy-Induced Hypertension[Title/Abstract])) OR (Pregnancy Induced Hypertension[Title/Abstract])) OR (Hypertensions, Pregnancy Induced[Title/Abstract])) OR (Induced Hypertension, Pregnancy[Title/Abstract])) OR (Induced Hypertensions, Pregnancy[Title/Abstract])) OR (Gestational Hypertension[Title/Abstract])) OR (Hypertension, Gestational[Title/Abstract])) OR (Transient Hypertension, Pregnancy[Title/Abstract])) OR (Hypertension, Pregnancy Transient[Title/Abstract])) OR (Pregnancy Transient Hypertension[Title/Abstract])) OR (Eclampsia[Title/Abstract])) OR (Pre-Eclampsia[Title/Abstract])) OR (Hypertensive Disorders, Pregnancy[Title/Abstract])) OR (preeclampsia[Title/Abstract])) OR (((((((Diabetes, Gestational[MeSH Terms]) OR (Diabetes, Pregnancy-Induced[Text Word])) OR (Diabetes, Pregnancy Induced[Text Word])) OR (Pregnancy-Induced Diabetes[Text Word])) OR (Gestational Diabetes[Text Word])) OR (Diabetes Mellitus, Gestational[Text Word])) OR (Gestational Diabetes Mellitus[Text Word]))**	**(Ethiopia[MeSH Terms]) OR (Federal Democratic Republic of Ethiopia[Text Word])**	**(((pregnant women[MeSH Terms]) OR (Pregnant Woman[Text Word])) OR (Women, Pregnant[Text Word])) OR (Woman, Pregnant[Text Word])**
25.		**((((((((((((((((((Hypertension, Pregnancy-Induced[MeSH Terms]) OR (Hypertension, Pregnancy**		

		**Induced[Title/Abstract])) OR (Pregnancy-Induced Hypertension[Title/Abstract])) OR (Pregnancy Induced Hypertension[Title/Abstract])) OR (Hypertensions, Pregnancy Induced[Title/Abstract])) OR (Induced Hypertension, Pregnancy[Title/Abstract])) OR (Induced Hypertensions, Pregnancy[Title/Abstract])) OR (Gestational Hypertension[Title/Abstract])) OR (Hypertension, Gestational[Title/Abstract])) OR (Transient Hypertension, Pregnancy[Title/Abstract])) OR (Hypertension, Pregnancy Transient[Title/Abstract])) OR (Pregnancy Transient Hypertension[Title/Abstract])) OR (Eclampsia[Title/Abstract])) OR (Pre-Eclampsia[Title/Abstract])) OR (Hypertensive Disorders, Pregnancy[Title/Abstract])) OR (preeclampsia[Title/Abstract])) OR (((((((Diabetes, Gestational[MeSH Terms]) OR (Diabetes, Pregnancy-Induced[Text Word])) OR (Diabetes, Pregnancy Induced[Text Word])) OR (Pregnancy-Induced Diabetes[Text Word])) OR (Gestational Diabetes[Text Word])) OR (Diabetes Mellitus, Gestational[Text Word])) OR (Gestational Diabetes Mellitus[Text Word]))) AND ((Ethiopia[MeSH Terms]) OR (Federal Democratic Republic of Ethiopia[Text Word]))) AND ((((pregnant women[MeSH Terms]) OR (Pregnant Woman[Text Word])) OR (Women, Pregnant[Text Word])) OR (Woman, Pregnant[Text Word]))**
26.		Limit to observational study and available full text articles
27.		34 studies

Cochrane Library

No.		Condition (Co);<br>Pregnancy Induced Hypertension<br>Diabetes, Gestational	Context (Co);<br>Ethiopia	Population (Po);<br>Pregnant Women
1.	**Mesh term**	**Hypertension, Pregnancy-Induced**	**Ethiopia**	**pregnant women**
2.		**Diabetes, Gestational**		
3.	**Entry terms**	Hypertension, Pregnancy Induced	Federal Democratic Republic of Ethiopia	Pregnant Woman
4.		Pregnancy-Induced Hypertension		Women, Pregnant
5.		Pregnancy Induced Hypertension		Woman, Pregnant
6.		Hypertensions, Pregnancy Induced		
7.		Induced Hypertension, Pregnancy		
8.		Induced Hypertensions, Pregnancy		
9.		Gestational Hypertension		
10.		Hypertension, Gestational		
11.		Transient Hypertension, Pregnancy		
12.		Hypertension, Pregnancy Transient		
13.		Pregnancy Transient Hypertension		
14.		Eclampsia		
15.		Pre-Eclampsia		
16.		Preeclampsia		
17.		Hypertensive Disorders, Pregnancy		
18.		Diabetes, Pregnancy-Induced		
19.		Diabetes, Pregnancy Induced		
20.		Pregnancy-Induced Diabetes		
21.		Gestational Diabetes		
22.		Diabetes Mellitus, Gestational		
23.		Gestational Diabetes Mellitus		
24.		**(Hypertension, Pregnancy Induced OR Pregnancy-Induced Hypertension OR Pregnancy Induced Hypertension OR Hypertensions, Pregnancy Induced OR Induced Hypertension, Pregnancy OR**	**(Ethiopia OR Federal Democratic Republic of Ethiopia):ti,ab,kw**	**(pregnant women OR Pregnant Woman OR Women, Pregnant OR Woman, Pregnant Word**

		**Induced Hypertensions, Pregnancy OR Gestational Hypertension OR Hypertension, Gestational OR Transient Hypertension, Pregnancy OR Eclampsia OR Pre-Eclampsia OR Preeclampsia OR Hypertensive Disorders, Pregnancy) OR (Diabetes, Gestational OR Gestational Diabetes OR Diabetes, Pregnancy Induced OR Gestational Diabetes Mellitus OR Pregnancy-Induced Diabetes OR Diabetes Mellitus, Gestational):ti,ab,kw**		**Women, Pregnant OR Woman, Pregnant):ti,ab,kw**
25.		**#1**	**#2**	**#3**
26.		**(#1 AND #2 AND #3)**		
27.		**2 studies**		

Science Direct

Mnemonic	Searching query
Condition (Co); Pregnancy Induced Hypertension	“Pregnancy Induced Hypertension" AND “Diabetes Mellitus” AND "Pregnant Women" AND "Ethiopia"
Context (Co); Ethiopia	
Population (Po); Diabetic Pregnant Women	

Number of Studies	Activities	
95	Total studies	without filtration
12	Only Research articles	With filtration

Embase

Number of Studies	Activities	
27	Total studies	without filtration
5	Open access and open archive	With filtration
	Only Research articles	

Google scholar

Searching Databases	Number of Studies	Activities	
**Google Scholar**	10	Total studies	With filtration

Overall refined studies

Activities		Number of Studies
Total studies have been selected (n=168)	PubMed	34
	Cochrane	2
	Science Direct	95
	Embase	27
	Google Scholar	10
	Total	168
Study selection (n=94)	Un-available full-text available (n=3)	94
	Duplicated record (20)	
	Articles other than observational study (n=71)	
Final exported studies to JBI		74
