# Supplementary material for: Double burden of gestational diabetes and pregnancy-induced hypertension in Ethiopia: A systematic review and meta-analysis of observational studies
Source: PLoS One. 2024 Oct 2;19(10):e0311110. doi: 10.1371/journal.pone.0311110 (PMC11446441; doi:10.1371/journal.pone.0311110)
Supplement: S2 Appendix — (PDF) [file pone.0311110.s012.pdf]

S.No	Study	Status	Reason for exclusion	URL
1.	Asefa 2020	Excluded	Outcome variables did not meet our inclusion criteria	http://198.1.99.189/index.php/ejrh/article/view/397
2.	Boka 2019	Included		https://www.longdom.org/open-access/assessment-of-adverse-birth-outcomes-and-associated-factors-among-diabetic-pregnant-mothers-who-delivered-at-mettu-karl-hospital-i-31825.html
3.	Eshetu 2019	Included		https://onlinelibrary.wiley.com/doi/full/10.1155/2019/6942617
4.	Andarge 2020	Included		https://www.longdom.org/open-access/prevalence-and-associated-factors-of-preeclampsia-among-pregnant-women-at-antenatal-booking-in-the-halaba-kullito-genera.pdf
5.	Ayalew 2019	Included		https://www.researchsquare.com/article/rs-2457/v1
6.	Baynesagn 2018	Excluded	Outcome variables did not meet our inclusion criteria	http://ir.haramaya.edu.et/hru/bitstream/handle/123456789/3219/Semagn%20Alemnew.pdf?sequence=1
7.	Belay 2019	Excluded	Outcome variables did not meet our inclusion criteria	https://link.springer.com/article/10.1186/s40885-019-0120-1
8.	Firisa 2021	Included		https://www.iprjb.org/journals/index.php/JHMN/article/view/1365
9.	Debele 2023	Included		https://assets-eu.researchsquare.com/files/rs-2523810/v1/71c7b596-1240-475e-8e0e-2da0eaf5e6ba.pdf?c=1680066883
10.	Duko 2021	Included		https://onlinelibrary.wiley.com/doi/full/10.1155/2021/6947499
11.	Haymanot 2020	Included		http://info.submit4journal.com/id/eprint/545/
12.	Hinkosa 2020	Excluded	Outcome variables did not meet our inclusion criteria	https://link.springer.com/article/10.1186/s12884-019-2693-9
13.	Kahsay 2018	Included		https://link.springer.com/article/10.1186/s12884-018-2106-5
14.	Katore. 2021	Included		https://www.tandfonline.com/doi/full/10.2147/IBPC.S336651
15.	Kidane 2022	Included		https://www.tandfonline.com/doi/full/10.2147/JMDH.S373749
16.	Wakwoya 2018	Included		https://www.ajol.info/index.php/tjog/article/view/178374
17.	Jikamo 2022	Excluded	Outcome variables did not meet our inclusion criteria	https://www.one-health.panafrican-medjournal.com/content/article/9/3/full/
18.	Birhanu 2020	Excluded	Outcome variables did not meet our inclusion criteria	https://www.tandfonline.com/doi/full/10.2147/IJWH.S265643

19.	Muche 2020	Included		https://link.springer.com/article/10.1186/s12884-020-2759-8
20.	Welesemayat 2020	Included		https://www.ajol.info/index.php/ejhd/article/view/201334
21.	Wolka 2022	Included		https://www.sciencedirect.com/science/article/abs/pii/S1871402121003842
22.	Berhe 2020	Excluded	Ineligible population	https://www.ajol.info/index.php/pamj/article/view/211266
23.	Wolie Asres 2023	Excluded	Ineligible population	https://www.tandfonline.com/doi/full/10.1080/08037051.2023.2255687#abstract
24.	Fikadu 2021	Excluded	Outcome variables did not meet our inclusion criteria	https://onlinelibrary.wiley.com/doi/full/10.1111/jch.14073
25.	Eyeberu 2023	Excluded	Unrelated topic and ineligible population	https://link.springer.com/article/10.1186/s12978-023-01716-7
26.	Sendeku 2021	Excluded	Unrelated topic and ineligible population	https://www.ajol.info/index.php/ahs/article/view/215214
27.	Taye 2022	Excluded	Outcome variables did not meet our inclusion criteria	https://www.tandfonline.com/doi/abs/10.1080/03007995.2022.2083399
28.	Mekie 2021	Excluded	Unrelated topic	https://link.springer.com/article/10.1186/s12884-021-03647-2
29.	Assefa 2021	Excluded	Unrelated topic	https://journals.plos.org/plosone/article?id=10.1371/journal.pone.0294482
30.	Getaneh 2021	Excluded	Unrelated topic	https://onlinelibrary.wiley.com/doi/full/10.1155/2021/6551526
31.	Mekie 2021	Excluded	Unrelated topic and duplicate study	https://pubmed.ncbi.nlm.nih.gov/35926064/
32.	Hassen 2022	Excluded	Unrelated topic	https://journals.plos.org/plosone/article?id=10.1371/journal.pone.0272165
33.	Asres 2021	Excluded	Unrelated topic	https://link.springer.com/article/10.1186/s12889-023-15794-6
34.	Sitot 2020	Excluded	Unrelated topic	https://pubmed.ncbi.nlm.nih.gov/35944014/
35.	Muche 2019	Excluded	Outcome variables did not meet our inclusion criteria	https://link.springer.com/article/10.1186/s12884-019-2492-3
36.	Walle 2022	Excluded	Unrelated topic	https://onlinelibrary.wiley.com/doi/full/10.1002/jcla.24305
37.	Argaw 2021	Excluded	Ineligible population	https://onlinelibrary.wiley.com/doi/full/10.1155/2021/6598944
38.	Belay 2020	Excluded	Outcome variables did not meet our inclusion criteria	https://journals.plos.org/plosone/article?id=10.1371/journal.pone.0230638
39.	Usuzaki 2021	Excluded	Ineligible study design	https://www.ncbi.nlm.nih.gov/pmc/articles/PMC8029745/
40.	Getahun 2023	Excluded	Outcome variables did not meet our inclusion	https://www.researchgate.net/profile/Genanew-Kassie-

			criteria	Getahun/publication/370444622_Prevalence_of_Pregnancy-Induced_Hypertension_and_Associated_Factors_among_Women_Receiving_Antenatal_Care_in_Addis_Ababa_Ethiopia/links/6450a2054af7887352502f58/Prevalence-of-Pregnancy-Induced-Hypertension-and-Associated-Factors-among-Women-Receiving-Antenatal-Care-in-Addis-Ababa-Ethiopia.pdf
41.	Gudeta 2019	Excluded	Outcome variables did not meet our inclusion criteria	file:///C:/Users/hp/Downloads/ajol-file-journals_449_articles_187185_submission_proof_187185-5329-475736-1-10-20190604.pdf
42.	Wolde 2011	Excluded	Outcome variables did not meet our inclusion criteria	https://www.ncbi.nlm.nih.gov/pmc/articles/PMC3275872/
43.	Mohammednur 2019	Excluded	Outcome variables did not meet our inclusion criteria	https://www.iosrjournals.org/iosr-jm/papers/Vol15-issue1/Series-1/D1501012228.pdf
44.	Walle 2017	Excluded	Outcome variables did not meet our inclusion criteria	https://www.sciencedirect.com/science/article/pii/S2210778918306871#s0085
45.	Tesfa 2020	Excluded	Ineligible study design	https://journals.plos.org/plosone/article?id=10.1371/journal.pone.0239048
46.	Getaneh 2020	Excluded	Ineligible study design	https://link.springer.com/article/10.1186/s13052-020-00926-0
47.	Zewdie 2023	Excluded	Outcome variables did not meet our inclusion criteria	https://papers.ssrn.com/sol3/papers.cfm?abstract_id=4318393
48.	Areda 2022	Excluded	Unrelated topic	https://onlinelibrary.wiley.com/doi/full/10.1002/hsr2.806
49.	Tessema 2015	Excluded	Outcome variables did not meet our inclusion criteria	https://link.springer.com/article/10.1186/s12884-015-0502-7
50.	Chemeda 2022	Excluded	Outcome variables did not meet our inclusion criteria	https://www.sciencedirect.com/science/article/pii/S2214139122000853
51.	Muche 2020	Excluded	Duplicate study	https://link.springer.com/article/10.1186/s12884-020-2759-8
52.	Thapa 2021	Excluded	Outcome variables did not meet our inclusion criteria	https://www.ncbi.nlm.nih.gov/pmc/articles/PMC9200039/
53.	Mengistu 2020	Excluded	Unrelated topic	https://link.springer.com/article/10.1186/s12872-020-01399-z
54.	Belay 2020	Excluded	Outcome variables did not meet our inclusion	https://journals.plos.org/plosone/article?id=10.1371/journal.pone.02306

			criteria	38
55.	Aburezq 2020	Excluded	Ineligible study context	https://www.sciencedirect.com/science/article/abs/pii/S2210778920300994
56.	Mehare 2018	Excluded	Unrelated topic	https://pubmed.ncbi.nlm.nih.gov/32411252/
57.	Wolie Asres 2023	Excluded	Duplicated study	https://www.tandfonline.com/doi/full/10.1080/08037051.2023.2255687
58.	Yemane 2021	Excluded	Outcome variables did not meet our inclusion criteria	https://link.springer.com/article/10.1186/s12884-021-03712-w
59.	Fakharunissa 2022	Excluded	Ineligible study context	https://pjmhsonline.com/index.php/pjmhs/article/view/1333
60.	Ayele 2022	Excluded	Outcome variables did not meet our inclusion criteria	https://www.ajol.info/index.php/ejhs/article/view/223242
61.	Meazaw 2020	Excluded	Ineligible study design	https://journals.plos.org/plosone/article?id=10.1371/journal.pone.0237476
62.	Haile 2022	Excluded	Unrelated topic	https://www.tandfonline.com/doi/full/10.1080/2331205X.2021.2022846
63.	Eshetu 2022	Excluded	Unrelated topic	https://www.frontiersin.org/journals/publichealth/articles/10.3389/fpubh.2022.888935/full
64.	Gebremedhin 2021	Excluded	Unrelated topic	https://onlinelibrary.wiley.com/doi/full/10.1155/2021/5254997
65.	Sitotaw 2018	Excluded	Unrelated topic	https://core.ac.uk/download/pdf/199938009.pdf
66.	Marbell 2019	Excluded	Ineligible study context	https://ugspace.ug.edu.gh/items/89979386-89bb-4227-8a6b-29a002e687ba
67.	Tessema 2021	Excluded	Outcome variables did not meet our inclusion criteria	https://onlinelibrary.wiley.com/doi/full/10.1155/2021/7430827
68.	Behaile Teklemariam 2022	Excluded	Unrelated topic	https://www.jogcr.com/article_697338.html
69.	Figa 2021	Excluded	Outcome variables did not meet our inclusion criteria	https://www.sciencepublishinggroup.com/article/10.11648/j.ijbecs.20210703.14
70.	Taye 2022	Excluded	Outcome variables did not meet our inclusion criteria	https://www.tandfonline.com/doi/abs/10.1080/03007995.2022.2083399
71.	Meazaw 2020	Excluded	Duplicated study	https://journals.plos.org/plosone/article?id=10.1371/journal.pone.02374

				76
72.	Sitotaw 2018	Excluded	Duplicated study	https://www.sciencedirect.com/science/article/abs/pii/S2210778917304713
73.	Mersha 2019	Excluded	Ineligible study design	https://link.springer.com/article/10.1186/s12884-019-2617-8
74.	Mulualem 2019	Excluded	Ineligible study design	https://link.springer.com/article/10.1186/s13104-019-4128-0
75.	Mareg 2020	Excluded	Outcome variables did not meet our inclusion criteria	https://www.tandfonline.com/doi/full/10.2147/IJWH.S251342
76.	Kibret 2019	Excluded	Ineligible study design	https://www.cambridge.org/core/journals/public-health-nutrition/article/maternal-dietary-patterns-and-risk-of-adverse-pregnancy-hypertensive-disorders-of-pregnancy-and-gestational-diabetes-mellitus-and-birth-preterm-birth-and-low-birth-weight-outcomes-a-systematic-review-and-metaanalysis/D5B3EE64A0F1EF22F468947032262960
77.	Hinkosa 2020	Excluded	Duplicated study	https://link.springer.com/article/10.1186/s12884-019-2693-9
78.	Kahsay 2018	Excluded	Duplicated study	https://link.springer.com/article/10.1186/s12884-018-2106-5
79.	Sisay 2020	Excluded	Ineligible study design	https://journals.plos.org/plosone/article?id=10.1371/journal.pone.0240382
80.	Kidane 2022	Excluded	Duplicated study	https://www.tandfonline.com/doi/full/10.2147/JMDH.S373749
81.	Ayele 2020	Excluded	Ineligible study design	https://journals.sagepub.com/doi/full/10.1177/2050312120935471
82.	Zeru 2021	Excluded	Ineligible study design	https://www.nature.com/articles/s41598-021-01256-9
83.	Demissie 2022	Excluded	Outcome variables did not meet our inclusion criteria	https://www.sciencedirect.com/science/article/abs/pii/S2210778921005523
84.	Berhan 2016	Excluded	Ineligible study design	https://www.ajol.info/index.php/ejhs/article/view/132889
85.	Zegeye 2018	Excluded	Ineligible study design	https://link.springer.com/article/10.1186/s12884-018-2101-x
86.	Wakwoya 2018	Excluded	Duplicated study	https://www.ajol.info/index.php/tjog/article/view/178374
87.	Muchie 2020	Excluded	Ineligible study design	https://link.springer.com/article/10.1186/s12884-020-03271-6
88.	Katore. 2021	Excluded	Duplicated study	https://www.tandfonline.com/doi/full/10.2147/IBPC.S336651
89.	Desta 2022	Excluded	Ineligible study design	https://www.sciencedirect.com/science/article/pii/S2214139122000117

90.	Mogos 2017	Excluded	Ineligible study design	https://link.springer.com/article/10.1007/s10903-016-0410-6
91.	Nigatu 2012	Excluded	Ineligible study design	https://onlinelibrary.wiley.com/doi/abs/10.1111/j.1753-0407.2011.00181.x
92.	Kinshella 2021	Excluded	Ineligible study design	https://www.sciencedirect.com/science/article/pii/S2161831322005130
93.	Abate 2021	Excluded	Ineligible study design	https://www.sciencedirect.com/science/article/pii/S2405857221000073
94.	Bereda 2021	Excluded	Outcome variables did not meet our inclusion criteria	https://www.sciencepublishinggroup.com/article/10.11648/j.ijpc.20210703.11
95.	Ayenew 2021	Excluded	Ineligible study design	https://link.springer.com/article/10.1186/s12978-021-01103-0
96.	Belete 2020	Excluded	Ineligible study design	https://www.tandfonline.com/doi/full/10.2147/IDR.S250654
97.	Ali 2018	Excluded	Ineligible study design	https://www.researchgate.net/publication/328043592_Factors-affecting-the-utilization-of-antenatal-care-among-pregnant-women-a-literature-review
98.	Abraham 2022	Excluded	Ineligible study design	https://www.mdpi.com/2073-4409/11/9/1548
99.	Hall 2011	Excluded	Ineligible study design	https://link.springer.com/article/10.1186/1471-2458-11-564
100.	Atlaw 2021	Excluded	Ineligible study design	https://journals.sagepub.com/doi/full/10.1177/20503121211031126
101.	Shiferaw 2021	Excluded	Ineligible study design	https://journals.plos.org/plosone/article?id=10.1371/journal.pone.0245003
102.	Yalam 2018	Excluded	Ineligible study design	https://www.researchgate.net/publication/329040940_Risk_of_Pre-Eclampsia_in_Pregnant_Women_A_Scoping_Review#fullTextFileContent
103.	Dibaba 2013	Excluded	Ineligible study design	https://link.springer.com/article/10.1186/1742-4755-10-50
104.	Shaib 2017	Excluded	Ineligible study design	https://www.sciencedirect.com/science/article/abs/pii/B9780128035061000462
105.	Mengist 2021	Excluded	Ineligible study design	https://www.sciencedirect.com/science/article/pii/S221413912100055X
106.	Choudhury 2021	Excluded	Ineligible study design	https://www.sciencedirect.com/science/article/pii/S0753332221009677
107.	Ana 2021	Excluded	Ineligible study design	https://www.sciencedirect.com/science/article/abs/pii/S0889852921000426
108.	Abebe 2021	Excluded	Unrelated topic	https://www.sciencedirect.com/science/article/pii/S2214139124000015
109.	Endeshaw	Excluded	Outcome variables did not meet our inclusion criteria	https://www.sciencedirect.com/science/article/abs/pii/S0266613815000704
110.	Bune 2019	Excluded	Unavailable full text	https://www.sciencedirect.com/science/article/abs/pii/S1871402119304

				722
111.	Addisu 2021	Excluded	Outcome variables did not meet our inclusion criteria	https://www.sciencedirect.com/science/article/pii/S240584402100428X#sec3
112.	Samura 2012	Excluded	Ineligible study design	https://www.sciencedirect.com/science/article/abs/pii/S0020729212610273
113.	Bhutta 2014	Excluded	Ineligible study design	https://www.sciencedirect.com/science/article/abs/pii/S0140673614607923
114.	Shokri 2020	Excluded	Ineligible study design and ineligible study context	https://www.sciencedirect.com/science/article/pii/S2405844020306320
115.	Feleke 2021	Excluded	Outcome variables did not meet our inclusion criteria	https://link.springer.com/article/10.1186/s12905-021-01485-0
116.	Behboudi-Gandevani 2022	Excluded	Ineligible study design	https://www.frontiersin.org/journals/public-health/articles/10.3389/fpubh.2022.766943/full
117.	Bizuayehu 2022	Excluded	Unrelated topic	https://www.sciencedirect.com/science/article/abs/pii/S0266613822000869
118.	Gelaw 2022	Excluded	Unrelated topic	https://link.springer.com/article/10.1186/s12884-022-04892-9
119.	Yeshaw2020	Excluded	Ineligible study design	https://bmjopen.bmj.com/content/10/3/e034963.abstract
120.	Bizuayehu 2022	Excluded	Duplicated study	https://academic.oup.com/eurpub/article/31/4/776/6181981?login=false
121.	Tesfaye 2019	Excluded	Ineligible study design	https://www.cambridge.org/core/journals/public-health-nutrition/article/applying-international-guidelines-for-calcium-supplementation-to-prevent-preeclampsia-simulation-of-recommended-dosages-suggests-risk-of-excess-intake-in-ethiopia/2C088A2C3DD84C1D1F6E7E45656ECDEC
122.	Mamo 2022	Excluded	Unrelated topic	https://journals.plos.org/plosone/article?id=10.1371/journal.pone.0262619
123.	Asefa 2016	Excluded	Unrelated topic	https://link.springer.com/article/10.1186/s12978-016-0225-x
124.	Hailu 1994	Excluded	Unrelated topic	https://pubmed.ncbi.nlm.nih.gov/7821247/
125.	Belay Tolu 2020	Excluded	Outcome variables did not meet our inclusion criteria	https://journals.plos.org/plosone/article?id=10.1371/journal.pone.0230638
126.	Dese 2022	Excluded	Unrelated topic	https://pubmed.ncbi.nlm.nih.gov/35509911/
127.	Teka 2022	Excluded	Outcome variables did not meet our inclusion criteria	https://journals.sagepub.com/doi/full/10.1177/17455057221078739

128.	Tekola 2019	Excluded	Unrelated topic	https://link.springer.com/article/10.1186/s12884-021-03601-2
129.	Asaye 2023	Excluded	Ineligible study design	https://journals.plos.org/plosone/article?id=10.1371/journal.pone.0285280
130.	Feleke 2022	Excluded	Unrelated topic	https://bmjopen.bmj.com/content/12/9/e061061.abstract
131.	Gejo 2021	Excluded	Outcome variables did not meet our inclusion criteria	https://link.springer.com/article/10.1186/s12884-020-03503-9
132.	Dachew 2020	Excluded	Outcome variables did not meet our inclusion criteria	https://www.cambridge.org/core/journals/development-and-psychopathology/article/abs/hypertensive-disorders-of-pregnancy-and-the-risk-of-offspring-depression-in-childhood-findings-from-the-avon-longitudinal-study-of-parents-and-children/6AEC3C70A4AC27808180E4F79F06DC2D
133.	Kelemu 2020	Excluded	Outcome variables did not meet our inclusion criteria	https://www.mdpi.com/1422-0067/21/16/5837
134.	Kumbi 1999	Excluded	Unavailable full text	https://europepmc.org/article/med/10442112
135.	Mekbeb 1990	Excluded	Unavailable full text	https://europepmc.org/article/med/2307159
136.	Haile 2021	Excluded	Outcome variables did not meet our inclusion criteria	https://onlinelibrary.wiley.com/doi/full/10.1155/2021/4654828
137.	Beyene 2023	Excluded	Ineligible study design	https://eurjmedres.biomedcentral.com/articles/10.1186/s40001-023-01088-5
138.	Yimer 2020	Excluded	Ineligible study design	https://www.ejrh.org/index.php/ejrh/article/view/247
139.	Fikrie 2023	Excluded	Outcome variables did not meet our inclusion criteria	https://journals.hu.edu.et/hu-journals/index.php/ejmhs/article/view/881
140.	Tadese 2022	Excluded	Outcome variables did not meet our inclusion criteria	https://link.springer.com/article/10.1186/s12884-022-05021-2
141.	Seyoum 2021	Excluded	Unrelated topic	https://www.researchsquare.com/article/rs-605611/v1
142.	Mekuriaw 2021	Excluded	Outcome variables did not meet our inclusion criteria	https://www.jscimedcentral.com/jounal-article-info/JSM-Nutritional-Disorders/Dietary-Risk-Factors-of-Preeclampsia-among-Women-Attending-Antenatal-and-Delivery-Services-in-Governmental-Hospitals-of-West-Gojjam-Zone%2C-North-West-Ethiopia-11094
143.	Niyonzima 2021	Excluded	Ineligible study context	https://pubmed.ncbi.nlm.nih.gov/34308244/
144.	Asefa 2022	Excluded	Unrelated topic	https://bmjopen.bmj.com/content/12/6/e055660.abstract
145.	Harrison 2021	Excluded	Ineligible study design	https://www.ncbi.nlm.nih.gov/pmc/articles/PMC8136690/

146.	Asefa 2020	Excluded	Duplicated study	http://198.1.99.189/index.php/ejrh/article/view/397
147.	Boka 2019	Excluded	Duplicated study	https://www.longdom.org/open-access/assessment-of-adverse-birth-outcomes-and-associated-factors-among-diabetic-pregnant-mothers-who-delivered-at-mettu-karl-hospital-i-31825.html
148.	Eshetu 2019	Excluded	Duplicated study	https://onlinelibrary.wiley.com/doi/full/10.1155/2019/6942617
149.	Andarge 2020	Excluded	Duplicated study	https://www.longdom.org/open-access/prevalence-and-associated-factors-of-preeclampsia-among-pregnant-women-at-antenatal-booking-in-the-halaba-kullito-genera.pdf
150.	Ayalew 2019	Excluded	Duplicated study	https://www.researchsquare.com/article/rs-2457/v1
151.	Mossie 2022	Excluded	Ineligible study design	https://www.sciencedirect.com/science/article/pii/S2405857222000079
152.	Kanguru 2014	Excluded	Ineligible study design	https://www.tandfonline.com/doi/full/10.3402/gha.v7.23987
153.	Mogas 2021	Excluded	Outcome variables did not meet our inclusion criteria	https://www.tandfonline.com/doi/full/10.2147/IBPC.S293251
154.	Tesfaye 2019	Excluded	Ineligible study design	https://www.sciencedirect.com/science/article/abs/pii/S0168822719305406
155.	Gebre 2013	Excluded	Ineligible study design	https://www.ajol.info/index.php/ejhd/article/view/115324
156.	Jikamo 2022	Excluded	Duplicated study	https://www.one-health.panafrican-medjournal.com/content/article/9/3/full/
157.	Mersha 2019	Excluded	Duplicated study	https://link.springer.com/article/10.1186/s12884-019-2617-8
158.	Mulualem 2019	Excluded	Duplicated study	https://link.springer.com/article/10.1186/s13104-019-4128-0
159.	Mareg 2020	Excluded	Duplicated study	https://www.tandfonline.com/doi/full/10.2147/IJWH.S251342
160.	Gupta 2019	Excluded	Ineligible study context	http://jamdsr.com/uploadfiles/33vol6issue9p128-131.20210420095611.pdf
161.	Dahir 2020	Excluded	Ineligible study context	https://www.sciencepublishinggroup.com/article/10.11648/j.cajph.20200606.12
162.	Bergdahl 2020	Excluded	Ineligible study context	https://gupea.ub.gu.se/handle/2077/64144
163.	Abban 2021	Excluded	Ineligible study context	https://ugspace.ug.edu.gh/items/00cc7a79-2339-4058-8021-e9421d9e232d

164.	Walle 2020	Excluded	Ineligible study design	https://www.tandfonline.com/doi/full/10.2147/NDS.S274646
165.	Fikadu 2020	Excluded	Outcome variables did not meet our inclusion criteria	https://link.springer.com/article/10.1186/s40885-020-00149-9
166.	Mengistu 2020	Excluded	Unrelated topic	https://link.springer.com/article/10.1186/s12872-020-01399-z
167.	Yimer 2020	Excluded	Ineligible study design	https://www.ejrh.org/index.php/ejrh/article/view/247
168.	Aburezq 2020	Excluded	Ineligible study context	https://www.sciencedirect.com/science/article/abs/pii/S2210778920300994
