# Supplementary material for: Double burden of gestational diabetes and pregnancy-induced hypertension in Ethiopia: A systematic review and meta-analysis of observational studies
Source: PLoS One. 2024 Oct 2;19(10):e0311110. doi: 10.1371/journal.pone.0311110 (PMC11446441; doi:10.1371/journal.pone.0311110)
Supplement: Attachment — Submitted filename: Response to Reviewers.pdf [file pone.0311110.s015.pdf]

Response to Editor and Reviewers

We sincerely appreciate the handling editor and reviewers for their time and insightful comments. As requested, we have revised our manuscript in accordance with the comments provided by the handling editor. Please find our responses below.

Journal Requirements:

Please review your reference list to ensure that it is complete and correct. If you have cited papers that have been retracted, please include the rationale for doing so in the manuscript text, or remove these references and replace them with relevant current references. Any changes to the reference list should be mentioned in the rebuttal letter that accompanies your revised manuscript. If you need to cite a retracted article, indicate the article's retracted status in the References list and also include a citation and full reference for the retraction notice.

- ***Authors' response:*** *Thank you for your feedback. We have reviewed and formatted all references according to the journal's requirements. If any references were missed or found to be inaccurate, we will be happy to update them.*

Additional Editor Comments:

I have seen the authors' response to the reviewers' and my comments, and they have addressed most of the issues. However, they should include a sensitivity analysis and comment on their findings by excluding case-control studies.

- ***Authors' response:*** *Thank you for the suggestion. Excluding case-control studies did not significantly alter the pooled prevalence of GDM/PIH co-occurrence among pregnant women in Ethiopia. We have updated the manuscript to reflect these results, along with the supporting figure.*
