# Supplementary material for: Double burden of gestational diabetes and pregnancy-induced hypertension in Ethiopia: A systematic review and meta-analysis of observational studies
Source: PLoS One. 2024 Oct 2;19(10):e0311110. doi: 10.1371/journal.pone.0311110 (PMC11446441; doi:10.1371/journal.pone.0311110)
Supplement: Attachment — Submitted filename: comment.docx [file pone.0311110.s013.docx]

Thank you for inviting me to review the manuscript on Double burden of pregnancy-induced hypertension and gestational diabetes mellitus among pregnant women in Ethiopia: a systematic review and meta-analysis. I think the title is very impressive and gives a good clue for scholars and policy makers. In general the research topic is interesting and it is researchable. I have some comments and suggestions for the author.

1. Title should be smart and should answer what, whom, when and what questions ; but your topic not answer the question when?
2. On abstract method section the period of study should be specified

**Introduction**

1. Line 72 you side that, there is limited evidence regarding the magnitude and correlation of PIH/GDM co-occurrence among pregnant women in Ethiopia; but there is around 15 original research evidences based on your reports; how did you see these controversy ideas?

**Method section**

Line 88 on Search strategy: you reported that you have used different search engines like PubMed, Cochrane Library, Science 88 Direct, Embase, HINARI, and Google Scholar; it is good to use such searching mechanisms ; to be clear you should report in each search engines terms, MUSH terms including the date of search and total number of result in each search clearly by table.

**Discussion**

**Your** discussion section is somewhat shallow; you should more explore the co-occurrence of GDM and PIH evidences, how the relations hypothesis from different evidences and then put your hypothesis.

**Recommendations**

1. **You should put recommendations separately to the conclusion**
2. **You should recommend specifically for the concerned bodies**
3. **The recommendation should be result based, specific, measurable and achievable**
