# Supplementary material for: Double burden of gestational diabetes and pregnancy-induced hypertension in Ethiopia: A systematic review and meta-analysis of observational studies
Source: PLoS One. 2024 Oct 2;19(10):e0311110. doi: 10.1371/journal.pone.0311110 (PMC11446441; doi:10.1371/journal.pone.0311110)
Supplement: Attachment — Submitted filename: Response to Reviewers.docx [file pone.0311110.s014.docx]

# Response to Editor and Reviewers

Dear editor Berhanu Elfu Feleke,

The authors would like to thank the editor and reviewers for their insightful comments and valuable feedback, which have significantly contributed to improving the quality of our paper. We have addressed all the comments and made the necessary revisions to enhance the manuscript. Thank you for your continued support and consideration.

# Additional Editor Comments

1. Format tables to the publication standards.

- ***Authors’ response:*** *Thank you for your feedback. We have formatted the tables to meet PLoS publication standards.*

2. How did you extract the prevalence data from case-control studies?

- ***Authors’ response:*** *Thank you for your insightful comment. The concern raised about pooling prevalence from such studies is valid, as it requires careful consideration to avoid misrepresenting the true burden of the conditions. To address this, we carefully aimed to ensure accurate representation. Excluding case-control studies would have limited our understanding of the overall burden. Therefore, we included prevalence data from these studies by recalculating the co-occurrence of GDM and PIH based on the entire population of the study, not just the case group. For example, in the study by Duko et al. (2021), with 283 participants (95 with PIH and 188 without), the prevalence of GDM among the PIH group was reported as 10.5%. To accurately reflect the burden of GDM/PIH co-occurrence, we recalculated the prevalence using the total sample size, resulting in a prevalence of 3.35%.*
- *Similarly, in the study by Haymanot et al. (2020), out of 200 participants (100 with PIH and 100 without), 8% of those with PIH had GDM. Again, by recalculating based on the total population, the co-occurrence rate was 4%. This method allowed us to provide a more comprehensive and accurate estimation of the double burden of GDM and PIH among pregnant women in Ethiopia.*

3. Can you provide the age-specific estimates?

- ***Authors’ response:*** *Thank you for your valuable feedback. We understand the importance of providing age-specific estimates for PIH/GDM co-occurrence. However, the available data from the included studies allowed us to report only the mean (SD) of age, which summarized the overall age distribution of the study populations. Unfortunately, due to the nature of the data from the included studies and the fact that they did not calculate the prevalence of PIH/GDM within different age groups, we are unable to stratify the prevalence estimates by specific age groups.*

4. Discuss the significance and implications of your findings to clinicians, public health experts, managers, decision/policy makers, researchers, and to pregnant women.

- ***Authors’ response:*** *Thank you for your suggestion. We have updated the manuscript in accordance with the given comment.*

5. Comment on the effects of different diagnostic criteria (diagnosed/undiagnosed GDM or PIH) across primary studies to your estimate.

- ***Authors’ response:*** *Thank you for the valuable suggestion. We have incorporated the feedback and updated the manuscript accordingly.*

6. Have a descriptive title to all figures and tables.

- ***Authors’ response:*** *Thank you for your feedback. We have made the adjustments accordingly.*

# Reviewer #1

1. Title should be smart and should answer what, whom, when and what questions; but your topic not answer the question when?

- ***Authors’ Response:*** *Thank you for your insightful feedback. While we understand the importance of addressing the "when" aspect in research titles, we believe that including a specific time frame is not essential in this case due to the nature of systematic reviews, which synthesize data across various time periods. Our focus is on the overall prevalence and co-occurrence of diabetes and hypertension in Ethiopia, rather than a specific time frame. However, the "when" question is addressed in the search strategy section, where we included studies published up to May 14. Therefore, we have revised the title to “Double burden of gestational diabetes and hypertension in Ethiopia: A systematic review and meta-analysis of observational studies” to better reflect the study's scope and intent.*

2. On abstract method section the period of study should be specified

- ***Authors’ response:*** *Thank you for your suggestion. We have updated the abstract section accordingly.*

3. Introduction: Line 72 you said that, there is limited evidence regarding the magnitude and correlation of PIH/GDM co-occurrence among pregnant women in Ethiopia; but there is around 15 original research evidences based on your reports; how did you see these controversy ideas?

***Authors’ response:*** *Thank you for highlighting this point. While it is true that our review included 15 original research studies, the limited evidence we referred to concerns the comprehensive understanding and synthesis of the magnitude and correlation of PIH and GDM co-occurrence in Ethiopia. To clarify this and address any potential confusion, we have updated the statement as follows: "Despite the existence of some studies, there remains a lack of comprehensive evidence regarding the precise magnitude and correlation of PIH/GDM co-occurrence among pregnant women in Ethiopia.”*

4. Method section: Line 88 on Search strategy: you reported that you have used different search engines like PubMed, Cochrane Library, Science 88 Direct, Embase, HINARI, and Google Scholar; it is good to use such searching mechanisms; to be clear you should report in each search engines terms, MESH terms including the date of search and total number of result in each search clearly by table.

- ***Authors’ response:*** *Thank you for your feedback. We previously uploaded the search strategy details; however, we have now adjusted it as per the suggestion. The updated information, including specific search terms, MeSH terms, search dates, and the total number of results for each database, has been uploaded.*

5. Discussion: Your discussion section is somewhat shallow; you should more explore the co-occurrence of GDM and PIH evidences, how the relations hypothesis from different evidences and then put your hypothesis.

- ***Authors’ response:*** *Thank you for your suggestion. We have updated the discussion section accordingly.*

6. You should put recommendations separately to the conclusion

- ***Authors’ response:*** *We highly value the reviewer’s suggestions. To stick with the publication standards, we kept the recommendation statements under the conclusion but as separate sentences.*

7. You should recommend specifically for the concerned bodies

- ***Authors’ response:*** *Thank you for your feedback. Our recommendation statements in the previous version “This highlights the importance of routine screening for GDM and PIH early in pregnancy for all pregnant women (for Healthcare workers). Regular monitoring of blood glucose levels and blood pressure throughout pregnancy is essential to track changes and identify any emerging issues promptly (for Healthcare workers). Effective interventions are also needed to improve maternal health outcomes and reduce healthcare burden (for Public health, managers, and policy makers), while further research is warranted to understand underlying factors and evaluate intervention strategies (for Researchers)” have implicitly addressed the concerned stakeholders..*

8. The recommendation should be result based, specific, measurable and achievable

- ***Authors’ response:*** *Thank you for your feedback. We have updated the recommendations under the conclusion section to ensure they are specific, measurable, and achievable. The revised recommendations now target key stakeholders, including clinicians, public health experts, managers, decision/policy makers, and researchers.*

# Reviewer #2

1. What was the definition of GDM and PIH in this review? Were they consistent across studies?

- ***Authors’ response:*** *Thank you for your observation. In this review, the definition of GDM was consistently applied across all included studies as "Any degree of glucose intolerance that occurs during pregnancy, characterized by high blood glucose levels that were either absent or well-controlled before pregnancy."*
- *For PIH, the categorization included gestational hypertension (GH), pre-eclampsia, and eclampsia. However, there was variability in how PIH was reported across studies. Some studies used the term PIH broadly, while others specified pre-eclampsia or eclampsia. To ensure comprehensive reporting and avoid underestimating the burden of PIH, we adopted a broad classification approach in our outcome measurements. Studies reporting outcomes related to these conditions, whether alone or in combination, were included under PIH for the purpose of this review. This approach allowed us to include studies with different terminologies and maintain a more inclusive assessment of the prevalence and association of GDM and PIH.*
- *We have addressed this concern on page 4, lines 117-120, under sub heading “Outcome measurements”.*

2. Can the authors please explain how they obtained the data in Table 1? For example, how do they calculate prevalence from a case control study?

- ***Authors’ response:*** *Thank you for your insightful comment. The concern raised about pooling prevalence from such studies is valid, as it requires careful consideration to avoid misrepresenting the true burden of the conditions.*
- *To address this, we took a meticulous approach to ensure accurate representation. Excluding case-control studies would have limited our understanding of the overall burden. Therefore, we included prevalence data from these studies by recalculating the co-occurrence of GDM and PIH based on the entire population of the study, not just the case group.*
- *For example, in the study by Duko et al. (2021), with 283 participants (95 with PIH and 188 without), the prevalence of GDM among the PIH group was reported as 10.5%. To accurately reflect the burden of GDM/PIH co-occurrence, we recalculated the prevalence using the total sample size, resulting in a prevalence of 3.35%.*
- *Similarly, in the study by Haymanot et al. (2020), out of 200 participants (100 with PIH and 100 without), 8% of those with PIH had GDM. Again, by recalculating based on the total population, the co-occurrence rate was 4%.*
- *This method allowed us to provide a more comprehensive and accurate estimation of the double burden of GDM and PIH among pregnant women in Ethiopia.*

3. Furthermore, for many of the papers, the data used to populate Table 1 were not immediately evident in the references. Add study nos to the references in Table 1

- ***Authors’ response:*** *Thank you for your insightful comment. We have updated the table accordingly.*

4. Some of references. for example, Duko (is this Belayhun?), Wolka, Katore and Muche are not in reference list

- ***Authors’ response:*** *Thank you for your comment. The reference labeled as "Duko" in Table 1 refers to the author listed in the Belayhun paper. This naming appeared as "Duko" during the exportation process from EndNote to JBI for critical appraisal.*
- *We have included all the references in the table as per your suggestion.*

5. Use decimals consistently

- ***Authors’ response:*** *Thank you for your feedback. We have reviewed the manuscript and ensured that decimals are used consistently throughout the document, including in the table.*

6. Did the authors distinguish between gestational diabetes and type1/type 2 diabetes?

- ***Authors’ response****: Yes, we clearly distinguished between gestational diabetes mellitus (GDM) and pre-existing type 1 or type 2 diabetes. We outlined this distinction in the outcome measurements section, specifying that pregnant women with pre-existing diabetes or hypertension were excluded from the study. As stated on page 4, lines 119-120, our review focused solely on outcomes related to GDM and PIH, ensuring that only studies pertaining to gestational conditions were included.*

7. In the introduction, BMI is used without being written out

- ***Authors’ response:*** *Thank you for your observation. We have corrected this in the manuscript by writing out "Body Mass Index (BMI)" in full the first time it appears in the introduction.*

8. On page 7, not sure what 25 and 26 in brackets refer to? Also, 29 and 25 in the last line

- ***Authors’ response****: Thank you for pointing this out. The numbers in brackets refer to specific studies cited in the reference list. For instance, reference 25 had the lowest mean age of 25.6 (±4.8), while reference 26 had the highest mean age of 33.53 (±5.83). Additionally, reference 29 corresponds to the study with the smallest sample size of 128, whereas reference 25 had the largest sample size of 1,834. The inclusion of reference numbers within the tables will clarify these points in the revised manuscript.*
- *To avoid confusion, we have changed the citation style from parentheses ( ) to square brackets [ ].*

9. Prevalence could be affected by the diagnostic criteria used, setting (high risk (hospital) or clinic). Hospitals represent high risk populations, therefore, is not reflective of the burden in Ethiopia? This could also explain the discrepancies with other studies in the discussion.

- ***Authors’ response****: Thank you for your comment. We have updated the manuscript accordingly to reflect this consideration.*

10. Population-based studies are preferable to assess prevalence; the authors need to include the importance of study designs in the discussion.

- ***Authors’ response****: Thank you for the insightful suggestion. We have updated the manuscript accordingly.*

11. Do not use it's rather write out as it is

- ***Authors’ response****: Thank you for the comment. We have updated the manuscript accordingly.*

12. For the subgroup analysis by sample size, why was 300 selected?

- ***Authors’ response****: Thank you for your comment. The choice of a sample size of 300 for the subgroup analysis was based on established guidelines for ensuring adequate statistical power and reliable results. Research suggests that a minimum sample size of 200 is generally sufficient for robust data analysis (Hoe, 2008; Singh et al., 2016). However, a sample size of 300 is recommended for achieving better stability in factor analysis and is considered "good" according to Comrey and Lee's criteria (Comrey & Lee, 1992; Tabachnick & Fidell, 2013). This threshold helps ensure that our subgroup analysis is sufficiently powered to detect meaningful effects and improve the robustness of our findings.*

13. Comment on the quality of the evidence and pooled estimate in the context of the limitations of study designs and reporting, and overall heterogeneity.

- ***Authors’ response:*** *Thank you for your feedback. We have updated the 'Strengths and Limitations of the Study' section of the manuscript accordingly based on your suggestion.*

14. How did this review limit publication bias?

- ***Authors’ response:*** *Thank you for your comment. To address potential publication bias, we implemented several strategies. We pre-registered our review protocol to ensure transparency and prevent selective reporting. Although we included studies published up to May 14, 2023, we conducted a comprehensive search strategy to identify both published and unpublished studies with no language restrictions. To ensure that no relevant articles were missed, we employed three reviewers for the selection process. Additionally, we adhered to PRISMA guidelines and assessed publication bias using Egger’s test, which indicated no significant bias. These measures were intended to minimize the influence of publication bias on our findings.*

15. Elaborate on the limitations of the systematic review with regard to study design, included studies, etc.

- ***Authors’ response****: Thank you for your comment. We have updated the manuscript in accordance with your suggestion.*
